# Mucosal Immunity and Sublingual Immunotherapy in Respiratory Disorders

**DOI:** 10.1155/2012/725719

**Published:** 2012-09-17

**Authors:** Nerin N. Bahceciler

**Affiliations:** Division of Allergy and Clinical Immunology, Pediatric Department, Near East University Hospital, Nicosia, North Cyprus, Turkey

The prevalence of allergic diseases, specially respiratory allergic diseases such as allergic rhinitis and asthma, has been increasing worldwide for the last 2 decades [1, 2]. Although avoidance of the responsible allergen, anti-inflammatory, and symptomatic treatment modalities has shown great efficacy in the treatment of allergic respiratory disorders, cessation of pharmacotherapy usually results in recurrence of signs and symptoms, with a demand to restart the treatment. 

Currently, allergen-specific immunotherapy (SIT) is the only available curative choice with the capacity of altering the natural course of allergy [3, 4]. Although SIT by the subcutaneous route has been extensively used and has shown marked efficacy since its discovery, it was associated with uncommon, but severe or even fatal, systemic reactions [5]. Consequently, alternative, noninjectve allergen delivery routes have been proposed, and allergen delivery through mucosal surfaces was suggested as a possible mechanism for the induction of mucosal tolerance to allergens [5, 6]. Local mucosal routes such as oral, nasal, bronchial, and sublingual were investigated since then, and controlled trials failed to demonstrate satisfactory clinical efficacy and/or safety of oral, nasal, and bronchial allergen application; therefore those routes have been abandoned [7–11]. Meanwhile, the efficacy and safety of SIT via the sublingual route was well documented by a number of controlled trials both in children and adults with asthma and/or rhinitis [12, 13]. Since then, sublingual immunotherapy (SLIT) in the liquid drop formulation has been tested in a large number of double-blinded, placebo-controlled studies, and those studies were included in Cochrane meta-analyses [14–16] demonstrating efficacy both in children and adults with allergic rhinitis or asthma sensitized to house dust mite or various pollens. Thereafter, orodispersible grass-pollen tablets were developed and recent well-designed, well-powered, double-blinded, placebo-controlled studies demonstrated efficacy and safety of tablet formulation [17–20].

Some of those studies improved our understanding of the underlying immunological mechanisms in addition to the proven safety and efficacy. Recent studies demonstrated that SLIT exerts its immune-suppressive effect through the induction of Treg cytokines such as IL-10 and TGF-beta [21, 22]. This effect starts on the uptake of allergen by oral mucosal Langerhans cells through high-affinity IgE receptors [6]. More recent studies demonstrated increase in expression of Foxp3+ cells in the sublingual mucosa, which was accompanied by the systemic immunologic response during SLIT [23]. 

Hereby in this issue, data on clinical implications, efficacy, compliance, monitorization of delivery, and immunological mechanisms of allergen SIT delivered by the mucosal-mainly sublingual route will be presented.

*Nerin N. Bahceciler* *Nerin N. Bahceciler*
